# Editorial: Resident and Ectopic FGF Signaling in Development and Disease

**DOI:** 10.3389/fcell.2020.00720

**Published:** 2020-08-26

**Authors:** Fen Wang, Xiaokun Li, Cong Wang

**Affiliations:** ^1^Texas A&M Health Science Center, Institute of Biosciences and Technology, College Station, TX, United States; ^2^Department of Translational Medicine, College of Medicine, Texas A&M University, College Station, TX, United States; ^3^School of Pharmaceutical Sciences, Wenzhou Medical University, Wenzhou, China

**Keywords:** fibroblast growth factor, receptor tyrosine kinase, intrinsic cell signaling, ectopic cell signaling, canonical signaling, non-canonical signaling

The fibroblast growth factor (FGF) family is one of the largest growth factor families, which consists of 18 receptor-binging, intrinsic tissue regulatory polypeptides that share similarity in structures and amino acid sequences (Mckeehan et al., 2009; Wang et al., 2013, 2019). The FGFs are broadly expressed, which regulate cell proliferation, differentiation, survival, and function. The FGFs elicit their regulatory activities by binding and activating the FGF receptor (FGFR) transmembrane tyrosine kinases encoded by four highly homologous genes. Beside the FGF and FGFR, the FGF signaling complex also includes highly diverse heparan sulfate (HS) polysaccharides and klothos as co-receptors that not only affect the ligand-binding activity, but also modulate signaling specificity of the FGFR. Binding of the FGF to the FGFR-HS complex changes the conformation of the complexes, leading to receptor autophosphorylation, as well as phosphorylation of downstream signaling molecules, and thus, transmits the signals to downstream effectors. Both FGF and FGFR are expressed in a highly spatiotemporally and cell type-specific manner. Ectopic expression of the FGF and FGFR isoforms has been identified as culprits for multiple diseases, including developmental defects, cancer, and metabolic disorders (Li et al., 2016; Wang et al., 2019).

As an intrinsic cell signaling axis, the FGF and FGFR are expressed almost ubiquitously in all tissues and stages, function through the autocrine, paracrine, or endocrine mechanisms. These intrinsic FGF signaling axes play important roles in embryonic development, adult tissue homeostasis, function, and regeneration. Redundant FGF and FGFR expression are common in multiple tissues and organs to warrant the signaling conveyance. Loss of FGF signaling has been reported as culprits for developmental disorders and many other diseases. However, when ectopically expressed, the FGF signaling axis is pathogenic in many cells and tissues, ranging from birth defects, metabolic disorders, to cancers and cardiovascular diseases. Multiple mechanisms have been attributed to ectopic FGF signaling, including ectopic expression of FGF, FGFR, and heparan sulfate proteoglycans (HSPG), gain-of-function mutations in the FGFR tyrosine kinases, and possibly other unidentified mechanisms.

Upon activation, FGFRs elicit both *canonical* and *non-canonical* signals. The *canonical* signals include FRS2α-dependent activation of ERK and PI3K, as well as FRS2α-independent activation of PLCγ (Zhang et al., 2008a,b, 2010, 2012a,b; Lin et al., 2011; Li et al., 2016; Wang et al., 2016, 2019); *non-canonical* signaling pathways include posttranslational modifications of lactate dehydrogenase A (LDHA) and transforming growth factor β-activated kinase 1 (TAK1), which increases the stability, as well as the activity of these enzymes (Zhang et al., 2004; Lan et al., 2008; Fan et al., 2011; Jin et al., 2017; Liu et al., 2018; Wang et al., 2018). Although FGFR1-4 are highly similar, share many common downstream pathways, and elicit similar *canonical* signals in some cells, they elicit opposite signals in many other cell types (Feng et al., 1997; Jin et al., 2003; Wang et al., 2019). The mechanism underlying this signaling specificity is poorly understood. Converging data showed that *non-canonical* signaling likely accounts for the receptor isoform-specific signals. However, how the four FGFRs elicit receptor-isoform signals still largely remains to be elucidated. This is partly due to the complexity of the FGF signaling axis, which includes multiple ligands, receptors, and cofactors, as well as poorly understood *non-canonical* downstream pathways that mediate receptor isoform-specific signals (Mckeehan et al., 2009; Wang et al., 2013; Li et al., 2016). Understanding how FGFR elicits receptor isoform-specific signals will provide new strategies for precision medicine for diseases that are caused by loss of intrinsic FGF signaling or gain of ectopic FGF signaling.

This Research Topic includes several review articles of important concepts related to how intrinsic FGF signaling controls stem cell self-renewal and differentiation, organ development, repair, and regeneration, how dysregulated FGF19-FGFR4 signaling axis contributes to hepatocellular carcinoma, as well as several original studies that reveal novel aspects of how intrinsic FGF signaling alleviates inflammation responses in the kidney and liver, how ectopic FGF signaling promotes inflammation in the liver and increases stemness of cancer stem cells, and how engineered FGF2 has improved therapeutic values by reducing the undesirable heparin-binding activity.

Stem cells play an important role in regenerative medicine. Understand how to regulate stem cells to remain dormant, or activate them to undergo self-renewal and differentiation will provide new therapeutic strategies for regenerative medicine. Mossahebi-Mohammadi et al. systematically reviewed the expression and function of FGF signaling pathway in stem cells, induced pluripotent stem cells, and epiblast-derived stem cells, and how it controlled the pluripotency and differentiation of these stem cells. Furthermore, they also discussed the cross talks of the FGF pathways with other signaling pathways, including transforming growth factor β, Wnt, and retinoic acid, to control stem cell self-renewal and differentiation.

Cancer stem cells are culprits for relapse of malignant cancer. Both *in vitro* and *in vivo* experiments show that spheroid-forming cancer stem cells have high tumorigenic activity. Quan et al. reported that the FGF-FGFR signaling axis is required for maintaining spheroid-forming *in vitro* and tumor-forming *in vivo* of pancreatic ductal adenocarcinoma cells. They further demonstrated that the FGF signals in these stem cells are mediated by the AKT pathway, which prevent SOX2 degradation and increase SOX2 nuclear localization. Inhibition of AKT activity impaired sphere-forming activity *in vitro* and tumor-forming activity *in vivo*. The authors also discussed how suppression of this signaling axis was of therapeutic value for pancreatic cancer.

FGF signaling plays an important role in embryonic development and organogenesis. The rudimentary digestive tract is initially a tube-like structure that is composed of epithelial cells surrounded by mesenchymal cells, which develops into distinct functional regions: the tongue, the pharynx, the esophagus, the stomach, the duodenum, the small intestine, the cecum, the large intestine, the colon, and the anus, as well as the pancreas and the liver. Lv et al. systematically reviewed how FGF10-FGFR2 signaling axis regulated the development of the digestive tract, including the taste papillae, tongue, salivary gland, stomach, pancreas, and liver. They also discussed how FGFR2 signaling regulated intestinal injury repair and the translational use of recombinant FGF7 and FGF10 to improves the short bowl syndrome, alleviated inflammatory bowel disease and ulcerative colitis disease.

As an endocrine FGF, FGF19 secreted from small intestine activates FGFR4 in the hepatocytes and regulates bile acid production and secretion (Yu et al., 2000, 2005; Wang et al., 2014). FGFR4 is an intrinsic FGFR in hepatocytes, it plays an important role in liver function and suppresses chemical-induced inflammation and tumorigenesis (Yu et al., 2002, 2003; Zhao et al., 2006; Huang et al., 2007, 2009; Luo et al., 2010). However, ectopic expression and activation of the FGF19-FGFR4 signaling axis is pathogenic. Liu et al. summarized the expression and signaling pathway of the FGF19-FGFR4 signaling axis, and how ectopic FGF19-FGFR4 signaling was associated with many types of cancer. Overactivated FGFR4 promoted proliferation and prevented apoptosis of hepatocellular carcinoma. Ectopically activation of FGFR4 was detected in breast cancer, head and neck squamous cell carcinoma, colorectal cancer and lung cancer. They also discussed how FGFR4 inhibition, either with small molecular inhibitor or neutralizing antibodies, could be used to treat cancer that overexpressed ectopic FGFR4.

Inflammation is a double-edged sword for the host. Overactive inflammation exacerbates injuries, including acute kidney injury and liver damage induced by concanavalin A. Tan et al. reported that treating the mice with FGF2 activated the FGFR signaling pathway in kidney cells both *in vitro* and *in vivo*, and reduced inflammatory reactions, and thereby, alleviated kidney damage. Furthermore, they also unraveled that the canonical FGF downstream pathways, ERK and AKT, were required for FGF2 to alleviate inflammation in kidney cells. Wang et al. also reported that human hepatic stellate cells expressed FGF21, FGFR1, and beta-Klotho (KLB). The expression of this intrinsic FGF signaling axis likely cross talked with the TNFα pathway. Overexpression of FGF21 in mice alleviated toxin-induced inflammation and damages in the liver. However, treating hepatic stellate cells with FGF1 that bound to FGFR1 independent of KLB activated the non-canonical TAK1-mediated TNFα pathway and exacerbated inflammation reaction in the cells. The results demonstrate that the intrinsic FGF21-FGFR1/KLB signaling axis and ectopic FGF1-FGFR1 elicit functional opposite signals with respect to regulating inflammation.

Many members of the FGF family have the heparan sulfate/heparin binding activity (Li et al., 2016). As a co-factor, heparan sulfates enhance the binding of FGF and FGFR. They also determine the receptor-binding profiles of FGF, as well as signaling profile of the FGFR, and stability of the FGF. Koledova et al. tested two engineered FGF2 that have low heparin-binding activities with respect to stability, heparin dependence, and dynamics of activating ERK. They found that the two FGF2 derivatives, FGF2-STAB1 and FGF2-STAB2, had a higher mitogenic activity than did the wildtype FGF2. They also induced ERK phosphorylation more potently and at a faster dynamic than wildtype FGF2. Since the engineered FGF2s do not bind to heparin and have a high activity in activating the canonical ERK pathway, they are of transitional values for activating the FGFR-ERK signaling pathway in the precision medicine (Li et al., 2016).

In summary, the FGF signaling is complicated, and in many cases, the signaling specificity and intensity is not only FGF and FGFR isoform-dependent, but also heparan sulfate and host cells-dependent. It can be either a foe or friend, and the only determinant of what they are is when, where, and how the signals are elicited. Yet, how resident FGF-FGFR signaling axis elicits canonical or non-canonical signals to regulate multiple cellular activities and how ectopic FGF signaling causes diseases largely remain an enigma. Solving this enigma is needed for developing FGF-based precision therapies to treat diseases caused by aberrant FGF signals.

## Author Contributions

FW, XL, and CW conceived and wrote the editorial. All authors contributed to the article and approved the submitted version.

## Conflict of Interest

The authors declare that the research was conducted in the absence of any commercial or financial relationships that could be construed as a potential conflict of interest.
